# Wharton’s Jelly Mesenchymal Stromal Cells and Derived Extracellular Vesicles as Post-Myocardial Infarction Therapeutic Toolkit: An Experienced View

**DOI:** 10.3390/pharmaceutics13091336

**Published:** 2021-08-26

**Authors:** Noelia Muñoz-Domínguez, Santiago Roura, Cristina Prat-Vidal, Joaquim Vives

**Affiliations:** 1 Servei de Teràpia Cel·lular, Banc de Sang i Teixits, Edifici Dr. Frederic Duran i Jordà, Passeig Taulat, 116, 08005 Barcelona, Spain; noeliamunozdominguez@gmail.com; 2 ICREC Research Program, Germans Trias i Pujol Health Science Research Institute, Can Ruti Campus, 08916 Badalona, Spain; sroura@igtp.cat; 3 Heart Institute (iCor), Germans Trias i Pujol University Hospital, Carretera de Canyet s/n, 08916 Badalona, Spain; 4 CIBER Cardiovascular, Instituto de Salud Carlos III, 28029 Madrid, Spain; 5 Faculty of Medicine, University of Vic-Central University of Catalonia, 08500 Vic, Spain; 6 Institut d’Investigació Biomèdica de Bellvitge—IDIBELL, 08907 L’Hospitalet de Llobregat, Spain; 7 Musculoskeletal Tissue Engineering Group, Vall d’Hebron Research Institute (VHIR), Universitat Autònoma de Barcelona, Passeig de la Vall d’Hebron 129-139, 08035 Barcelona, Spain; 8 Departament de Medicina, Universitat Autònoma de Barcelona, Passeig de la Vall d’Hebron 129-139, 08035 Barcelona, Spain

**Keywords:** biomanufacturing, cardiac tissue engineering, clinical translation, extracellular vesicles, mesenchymal stromal cells, myocardial infarction, Wharton’s jelly

## Abstract

Outstanding progress has been achieved in developing therapeutic options for reasonably alleviating symptoms and prolonging the lifespan of patients suffering from myocardial infarction (MI). Current treatments, however, only partially address the functional recovery of post-infarcted myocardium, which is in fact the major goal for effective primary care. In this context, we largely investigated novel cell and TE tissue engineering therapeutic approaches for cardiac repair, particularly using multipotent mesenchymal stromal cells (MSC) and natural extracellular matrices, from pre-clinical studies to clinical application. A further step in this field is offered by MSC-derived extracellular vesicles (EV), which are naturally released nanosized lipid bilayer-delimited particles with a key role in cell-to-cell communication. Herein, in this review, we further describe and discuss the rationale, outcomes and challenges of our evidence-based therapy approaches using Wharton’s jelly MSC and derived EV in post-MI management.

## 1. Background

Cardiovascular diseases remain the most common cause of mortality worldwide [1]. A long list of risks including sedentary lifestyle and obesity among other key factors are known to potentially harm de cardiovascular system [2]. Myocardial infarction (MI), caused by a lack of oxygen delivery to the myocardial tissue, is the most common cardiovascular disease which results in irreversible damage to the heart muscle that may impair cardiac function and lead to heart failure. Ultimately, heart transplantation is the last option to improve survival in end-stage heart failure although is hampered by the low number of organ donors and adverse allograft rejection [3]. Other limitations to reach complete heart function recovery include possible side effects of immune-suppressive drugs on other recipient’s organs and the need for extremely complex coordinated procedures in expensive healthcare facilities [4,5].

Over the past few decades, this clinical scenario was spurred by initiatives addressing the design, development and assessment of a number of cell-based therapies to increase cardiac function recovery following MI [6]. In this context, the first efforts using mixed or enriched bone marrow mononuclear cell populations were extremely inefficient due to low cell retention, survival, and differentiation rates once administered. Further experiences were focused on intracoronary or intramyocardial delivery of mesenchymal stromal cells (MSC), also from bone marrow, subcutaneous adipose tissue or umbilical cord. Unfortunately, in general, treatment outcomes after conventional cell therapy in humans have been modest so far, because of the difficulties involved in repairing usually large myocardial scars and the low efficacy of administered cells [7]. Alternatively, cardiac tissue engineering (TE) emerged as a new therapeutic modality combining reparative cells with supporting materials (either natural or synthetic) in a three-dimensional (3D) context, although their clinical application is still very limited [8]. In present times, another strategy involving the use of extracellular vesicles (EV), which are double-layered membrane nanovesicles secreted by most cells to their microenvironment, has gained interest. In particular, EV secreted from multipotent mesenchymal stromal cells (MSC) are considered a valuable alternative to MSC themselves because they are potentially more efficient in transferring specific molecular cargoes and associated functions to targeted cells or tissues without the difficult logistics and safety risks associated with cell therapy. Thus, MSC-secreted EV (MSC-EV) may be useful immune-modulatory, cardioprotective and angiogenic agents post-MI, as shown in vitro and in experimental animal models [9,10].

Herein, we review the scientific bases, current therapy toolkit and associated outcomes as well as the future challenges for the development of novel treatments using MSC-EV.

## 2. Foundations of an Advanced Post-Myocardial Infarction Therapy

### 2.1. Wharton’s Jelly Mesenchymal Stromal Cells: The Active Ingredient

Multipotent MSC are self-renewing, ex vivo culture-expandable stem cell populations that can be commonly collected from the stroma of almost all tissues and organs [11,12]. In regard to its origin, MSC arise most likely from the perivascular space [12]. According to the following minimal criteria established by the International Society for Cell and Gene Therapy (ISCT), MSC must show: (i) plastic-adherence under standard in vitro culture conditions; (ii) specific surface expression pattern including the presence of CD105, CD73 and CD90, and absence of CD45, CD14, CD79α and HLA-DR; and (iii) in vitro ability to differentiate into mesodermal cell lineages (i.e., osteogenic, adipogenic and chondrogenic) [13]. In addition to their multipotent differentiation capacity, MSC also exhibit marked immune modulation potential and, thus, they are considered immune-privileged [14,15,16,17].

Remarkably, the therapeutic potential of MSC lies in their capacity to secrete a myriad of paracrine factors into the microenvironment [18]. Mediators released by MSC are known to actively modulate diverse biological processes, including: (i) tissue regeneration and repair; (ii) progenitor cell differentiation; and (iii) immune/inflammatory responses [19]. In vivo, MSC are able to specifically migrate to damaged tissues, where they interact locally and regulate host reparative progenitors and/or immune cells (both from innate and adaptive immune systems) [20,21]. In this sense, MSC may induce functional changes of monocytes/macrophages, dendritic cells, T cells, B cells, and natural killer cells to regulate the overall immune system response [22]. This is, for instance, the case of MSC derived from the umbilical cord and the adipose tissue surrounding the human heart that equally inhibit the inflammatory response of stimulated T cells [10,23]. Despite the complexity of molecular pathways and immune cell types involved in immunologic disorders, the use of MSC for the treatment of patients with Graft versus Host Disease (GvHD) illustrates unequivocally their therapeutic potential, which can be further improved by generating pools of cells from different donors to ensure patient’s response [24,25,26]. Thus, therapies based on MSC and derivatives will be developed along with increasing progress in understanding their intrinsic mechanisms of action (MoA), and may also benefit from recent trends towards the generation of regulatory-approved, clinical-grade cell banks with homozygous human leukocyte antigen (HLA) haplotypes of high prevalence among populations at a global scale [27]. This strategy holds the potential to offer optimized, versatile, immune-compatible therapeutic cell products for allogeneic transplantation.

**Figure 1:**
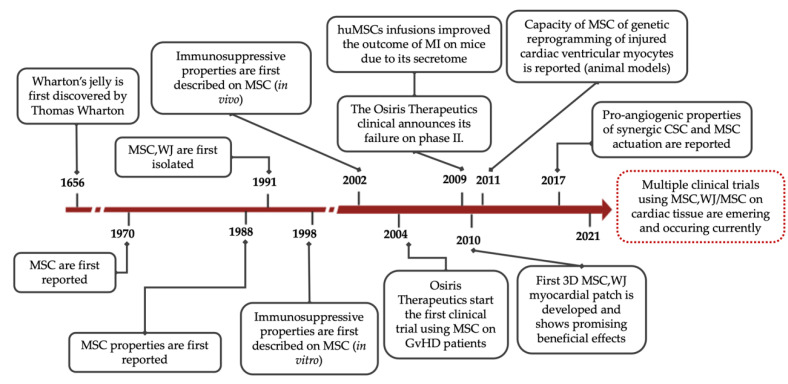
Timeline with major milestones in MSC research. MSC = Mesenchymal Stromal Cells; MSC,WJ = Wharton’s jelly-derived Mesenchymal Stromal Cells; CSC = Cardiac Stem Cells; GvHD = Graft-versus-Host Disease; MI = myocardial infarction.

Researchers already have the ability to readily isolate and scale-up large numbers of clinical-grade MSC from most tissue sources [11,16]. In particular, Wharton’s jelly (WJ), which is a gelatinous substance of connective tissue found in the umbilical cord donated after birth, is a plentiful source of MSC [28,29]. Historically, WJ was first described by Thomas Wharton back in 1656 [30], whereas McElreavey and collaborators reported the isolation of MSC,WJ in 1991 [31] (Figure 1). In terms of baseline characteristics, MSC,WJ are quite primitive cells with low risk to carry somatic mutations, thus are considered highly immune-privileged in comparison with other potential tissue sources. Moreover, clinical application of MSC is not restricted by either invasive, painful isolation procedures or intrinsic donor comorbidities (i.e., cardiovascular risk factors). MSC,WJ also exhibit high proliferation rates ex vivo, therefore allowing well-established, valuable, clinical-grade Master Cell and Working Cell Banks [16]. Notably, MSC,WJ express negligible levels of HLA-DR and low or null expression of the co-stimulatory molecules CD40, CD80 and CD86 [28,29]. Regarding HLA expression, we and others have previously demonstrated that HLA-DR expression is highly variable in primary MSC cultures, but it is almost undetectable in the case of MSC,WJ [28,32,33,34]. Additionally, their high secretion of inhibitory molecules such as PGE2 and the expression HLA-G6 isoform support the above-mentioned immune-privileged status by MSC,WJ [14]. Altogether, these data support the low probability of rejection and low toxicity of MSC,WJ once administered [35]. In this sense, in our laboratory, we confirmed that MSC,WJ are clinically useful and safe in the context of inflammatory conditions, including chronic spinal cord injury (EudraCT No. 2015-005786-23; ClinicalTrials.gov (accessed on 30 June 2021) Id. NCT03003364) and severe respiratory distress due to SARS-CoV-2 infection (EudraCT No. 2020-001505-22; ClinicalTrials.gov (accessed on 30 June 2021) Id. NCT04390139) [36,37]. 

### 2.2. Cardiac Extracellular Matrices: The Supportive Vehicles

Cardiac TE offers a plausible solution to overcome therapeutic limitations observed when reparative cells are delivered into the hypoxic infarcted area by either intracoronary administration or direct myocardial injection, thus increasing their cellular implantation and survival rates [38]. Furthermore, the use of supportive vehicles allows the incorporation of cells and/or bioactive factors for prolonged local retention and facilitates their biological activity or MoA. In brief, a variety of natural and synthetic materials have been used as cell supportive platforms generating engineered bioimplants or grafts that can be securely implanted over the post-infarcted heart [39]. Nevertheless, natural materials show enhanced biodegradable and biocompatible properties and can better recreate the native myocardium environment [40,41]. Particularly, the decellularized cardiac extracellular matrix (ECM) provides a close match to the native, physiological microenvironment with minor changes in stiffness while preserving the composition, vasculature network and 3D framework [42], while also enabling electromechanical coupling with the host myocardium after implantation [43,44]. For that, porosity and pore size are critical parameters for the functionality of decellularized scaffolds and determine their optimal mechanical properties, among other paramount factors [45]. Hence, the presence of open porous and interconnected networks is crucial to guarantee optimal cell nutrition, proliferation and migration for successful tissue repair and regeneration [45].

In our laboratory, a refined protocol for the manufacture of porous decellularized cardiac ECM from the human pericardium and porcine myocardium loaded with cardiac adipose tissue-derived MSC (MSC,CAT) was reported [46,47]. According to our observations, both decellularized porous scaffolds: (i) were optimal for accommodating host-derived cells; (ii) provided the necessary signalling cues to modulate cell function; and (iii) highly supported cell differentiation and survival [46,47]. However, proteome characterization of the two decellularized matrices showed enrichment of matrisome proteins and major cardiac ECM proteins, considerably higher for the recellularized pericardial graft. Moreover, although macro and micromechanics were well-maintained in both cardiac ECM following decellularization, the decellularized pericardial scaffold demonstrated improved cell infiltration and retention as well as larger pore size, making it the preferred scaffold for the biofabrication of solid organs or bioimplants [48,49]. Interestingly, decellularized ECM can be subjected to lyophilisation or sterilization procedures without significant mechanical changes, thus allowing their storage until use as off-the-shelf products for clinical use [49,50].

### 2.3. Evidence-Based Pre-Clinical Outcomes

In our laboratory, we collected robust in vivo data regarding the use of our two previously-described decellularized ECM in post-infarcted swine models. First, the neoformation of growing blood vessels and sprouting nerves in cardiac ECM made of decellularized pericardium once implanted in post-infarcted pigs suggested that: (i) both vascularization and innervation processes were supported by the ECM structure itself; (ii) were hypoxia-dependent; and (iii) required mobilization of host undifferentiated progenitor cells [49,50]. Second, implantation of cell-embedded cardiac bioimplants limited the sequelae associated with MI, particularly reducing infarct size and improving cardiac function. In these experiments, we specifically repopulated the decellularized human pericardial ECM by combining porcine MSC,CAT with the self-assembling peptide RAD16-I to generate optimal 3D conditions that efficiently promoted proliferation, maintained the differentiation commitment of MSC,CAT toward the endothelial lineage, and increased their migration from bioimplant to underlying injured myocardium [46]. Cardiac function was further assessed non-invasively by magnetic resonance imaging (MRI) and scar healing was evaluated by using a customized-design electrical impedance spectroscopy monitoring system incorporated within the bioimplant [51]. As a result, MRI detected a significant improvement in left ventricular ejection fraction (LVEF) and stroke volume in bioimplant-treated animals while morphometric measurements revealed a significant reduction in infarct size one month after implantation. Interestingly, we confirmed that noninvasive electrical impedance spectroscopy was useful for tracking differential scar healing, showing differences in impedance parameters between treated and control pigs. Indeed, myocardial tissue was preserved in bioimplant-treated animals, which was confirmed by histopathological measurements of reduced inflammation and altered collagen deposit [51].

Alternatively, administration of a similar ECM-based cardiac bioimplant combining decellularized porcine myocardial ECM refilled with porcine MSC,CAT also supported cardiac recovery in post-infarcted pigs. Our results reflected a higher improvement in LVEF after MI in the porcine myocardial ECM bioimplant-treated animals compared to those carrying the same cell-free scaffold [44] or other types of natural scaffolds [52,53]. Furthermore, engrafted bioimplant promoted revascularization of injured tissue, reduced infarct size, and attenuated ventricular remodeling and fibrosis progression [54].

A concluding comparison of functional benefits associated with the two decellularized ECM-based bioimplants was additionally reported by our group, as described in [49]. Additionally, decellularized scaffolds were either repopulated with porcine MSC,CAT or tested as cell-free scaffolds. Irrespective of the ECM origin or cell recolonization, both TE constructs were found well-integrated with the underlying myocardium and showed signs of neovascularization and nerve sprouting forty days after implantation. The combination of decellularized ECM scaffolds with MSC showed higher improvement than the cell-free scaffolds, indicating a synergistic effect of all bioimplant components in the therapeutic benefit of TE products [55]. Indeed, TE scaffolding may be beneficial for triggering MI recovery by providing a favorable microenvironment for the recruitment of endogenous progenitor cells towards the infarct bed by embedded MSC. The contribution of MSC to MI recovery has been previously reported [56], and paracrine signaling has been broadly described as one of the putative mechanisms by which implanted MSC can exert beneficial effects over the infarcted area [57,58].

Collectively, the presented evidence-based pre-clinical experience using MSC and cardiac ECM supports the achievement of beneficial effects on cardiac function following MI [59,60,61,62,63]. This was shown to be crucial for the regulatory approval of a novel advanced therapeutic medicinal product (ATMP) termed PeriCord by the Spanish Agency of Medicines and Medical Devices (AEMPS) (PEI18-140). PeriCord, which is composed of regulatory-approved MSC,WJ (PEI16-017) within decellularized pericardial ECM, potentially emerges as a new generation of TE-based treatment for MI. For that, its safety and efficacy are being evaluated in the clinical setting (Table 1) [64].

### 2.4. PeriCord: A Valuable CASE in Scalability and GMP Biomanufacturing of Cardiac Bioimplants

As previously mentioned, we explored the therapeutic potential of engineered cardiac bioimplants comprising cell-free cardiac scaffolds with preserved ECM structure and components aiming to deliver therapeutic MSC post-MI [66]. Remarkably, one of our two pre-clinically developed TE approaches has been scaled up to produce a clinical-size, good manufacturing practice (GMP)-compliant allogeneic ATMP. In specific, this novel ATMP is referred to as PeriCord and consists of ~16 cm^2^ cardiac bioimplant comprising clinical-grade MSC,WJ (the active ingredient) within human decellularized pericardial ECM (acting as a cell supportive material to facilitate surgical implantation). The acceptance criteria for initial PeriCord batch certification comprises: (i) a dose range of 7–15 × 10^6^ total viable MSC,WJ; (ii) cell viability ≥70%; and (iii) endotoxin ≤4 units/mL [50]. Safety data from PeriCord implantation are being evaluated in the ongoing phase I PERISCOPE (the PERIcardial matrix with mesenchymal Stem Cells fOr the treatment of PatiEnts with infarcted myocardial tissue) clinical trial (EudraCT No. 2018-001964-49; ClinicalTrials.gov (accessed on 30 June 2021) Id. NCT03798353) (Table 1). Importantly, eleven patients have already been recruite and no adverse effects directly related to the treatment have been observed to date.

In light of this clinical translation experience, we are currently taking further advantage of the window of opportunity that MSC-EV, instead of the MSC themselves, opens in terms of their plentiful cargo of molecules and associated functions, conserved morphology and integrity, and capacity of reaching either neighboring or distant cells and tissues upon administration. In particular, the rationale for the use of MSC-EV arises from the growing amount of data suggesting that these preparations are harmless and trigger, at least, similar effects to their parent cells. Moreover, MSC-EV are theoretically unaltered by microenvironmental factors due to their double-leaflet lipid membranes efficiently protect the inner molecular cargo from degradation and guarantee their entry into targeted cells. Additionally, their characteristic nanosize counteracts the potential risk of pulmonary thrombosis after intravascular administration of MSC due to the majority of infused cells are initially trapped in the lungs of recipients [67].

## 3. Mesenchymal Stromal Cell-Secreted Extracellular Vesicles: The Envisioned Alternative

In 1983, Stahl and collaborators reported that transferrin receptors were associated with small membranous vesicles that were actively expelled into the extracellular microenvironment by reticulocytes. This was one of the first descriptions of secreted cell-to-cell communication agents, which were later referred to as EV [68,69]. Notably, after nearly three decades of tremendous effort, EV are recognized as a wide diversity of lipid bilayer-delimited particles that are released by most cell types, including MSC. Succinctly, EV are distinctive in size, biogenesis, cargo molecules and function, and their classification is a major concern that remains controversial [70]. At present, EV are commonly divided into three categories according to size and formation pathway diversity as follows: (1) exosomes, which are intraluminal vesicular structures with a diameter ranging between 30 and 150 nm that are raised by the internal budding of the endosomal membrane during maturation of inner cellular multivesicular bodies (indeed, exosomes are increasingly designated “small EV”); (2) microvesicles that sprout directly from the plasma membrane and are released into the extracellular space, and have a wider size assortment than exosomes (50 nm–1 µm); and (3) apoptotic bodies, varying from 1 to 5 µm of diameter and externally released after an apoptotic cell disassembly procedure [71,72,73]. In an attempt to promote the standardization of EV characterisation, the International Society for Extracellular Vesicles (ISEV) proposed a set of “Minimal Information for Studies of Extracellular Vesicles” (or MISEV) guidelines for the field in 2014 and were recently updated in 2018 [73,74]. Indeed a better understanding of the composition of EV preparations may help to discern the actual biological activity of specific factors above the background.

Regarding their composition, EV contain a wide variety of bioactive compounds such as RNA species (mainly miRNA), lipids, and cytosolic proteins and transmembrane proteins in an appropriate and functional formulation, resembling the content of the parental cells. This has prompted the investigation of EV as useful blood-based biomarkers for disease diagnosis and prognosis, pharmaceutic targets of diseases, and active ingredients in the context of novel advanced cell-free therapies against cardiovascular conditions [10,75]. The content and functional attributes of EV depend on different conditions, including cell viability status, stage of activation, infection, stress, and neoplastic transformation, among others. For instance, the presence (or absence) of specific serum components clearly affects EV biogenesis and characteristics as one of the wide range of molecular changes that cells undergo in response to cellular stress [76]. In 2005, Savina and collaborators described EV secretion as highly dependent on the calcium handling machinery of the parental cells [77]. In addition, exogenously-added substances such as silver nanoparticles into the culture cell medium seem to promote EV formation and secretion [78]. However, exosomes can be restricted in therapies preparation due to their difficulties incorporating the specific cargo [79]. Collectively, these findings indicate that cells behave differently under stress conditions and therefore this may have an impact on the potential traits of their secreted EV, and point out the relevance of adjusting protocols for optimal cell culture conditions to guarantee the therapeutic efficacy of the resulting EV preparations.

Of note, EV may also act as an efficacious toolkit of cell-to-cell communication due to their ability to specifically modulate the molecular cargo and associated functions of targeted neighboring or distant cells or tissues [80]. In this regard, the protection conferred by these membranous nanovesicles to their internal effector molecules is crucial to warrant their triggered functions and governing MoA over time.

At the functional level, compelling pre-clinical studies show that MSC-EV are potent bioactive agents capable of modulating the host immune response, stimulating novel blood vessel formation (angiogenesis), cardioprotection (i.e., myocardial tissue injury reduction) and endothelial cell proliferation/migration, among other cardiovascular beneficial effects [81,82]. In brief, similar to the parental cells, EV have the potential to promote a shift in the pro-inflammatory milieu and functional changes in recipient immune cells, including monocytes/macrophages, dendritic cells, T cells, B cells and natural killer cells. In this context, the effect of MSC-EV on allogeneic T-cell stimulation and cytokine production in vitro has been found [83,84]. For instance, the addition of MSC-EV, such as those isolated from MSC,WJ using size-exclusion chromatography, was capable of powerfully preventing T-cell stimulation and reduced levels of adverse pro-inflammatory cytokine reaction [10].

In this context, MI and myocardial ischemia/reperfusion represent inflammation-associated diseases in which the immune-modulatory properties of MSC-EV could be of clinical relevance. Particularly, MI is accompanied by both exacerbated local and peripheral inflammatory responses, whereas myocardial ischemia/reperfusion triggers an over-activated inflammatory cascade in diseased hearts. However, in both conditions, the blockade of blood flow initiates an intense beneficial inflammatory effect that is essential for the early clearance of dead cells and subsequent cardiac repair and regeneration but, in turn, it becomes extremely deleterious if it is not timely suppressed. This leads to the post-infarction replacement of myocardial tissue by a non-contractile scar [85]. In this sense, MSC-EV seem to be valuable to modulate cardiac inflammation and improve overall cardiac functional parameters in failing hearts through distinct MoA that are currently under investigation. Numerous studies have shown that intramyocardial injection of MSC-EV from distinct tissue sources efficiently reduced the infarct size and enhances cardiac function preserving cardiac systolic and diastolic performance in ischemic rodent models [86,87,88,89]. In specific, it has been convincingly demonstrated that the benefit to macrophage polarization status is mediated by the miR-182 activity associated with MSC,WJ-secreted EV (MSC,WJ-EV) after their delivery in vivo [90]. In this same study of myocardial ischemia/reperfusion, intramyocardially injected MSC,WJ-EV also led to a remarkable reduction in infarct size and considerably alleviated undesirable inflammatory traits in both the heart and serum of EV-treated animals. Furthermore, engineered MSC-EV to overexpress miRNA-181a drastically influenced inflammatory response after myocardial ischemia-reperfusion injury, as demonstrated by Wei and collaborators [91]. These authors further confirmed that engineered MSC-EV led to a decrease in pro-inflammatory IL-6 and TNF-α levels, as well as an increase in anti-inflammatory cytokines such as IL-10 in injured mice. In order to allow these benefits, the mechanisms involved are considered multifactorial, since a joint action of antiapoptotic, anti-inflammatory and pro-survival effects happens [92].

In addition, administration of MSC-EV has shown to exert both protective and pro-regenerative effects against myocardial tissue damage provoked by acute MI, along with no risk of tumorigenicity and immune rejection after infusion [93].In fact, MSC-EV-driven cardio protection would include reduction in cardiomyocyte apoptosis and enhancement of cardiomyocyte viability post-MI. For instance, conditioned medium collected from cultured MSC and infused intravenously before reperfusion prompted a significant reduction in infarct size both in post-infarcted rodents and pigs [94]. Specifically, they concluded that benefit was reached by improved myocardial cell viability following in vivo treatment. This is in agreement with data from Arslan and collaborators, who observed that the administration of MSC-EV in mice resulted in increased ATP levels, decreased oxidative stress, and also triggered protective PI3K/Akt-mediated signaling in ischemic/reperfused hearts [95]. Furthermore MSC-EV delivery was capable of preventing cardiac muscle cells from apoptosis, and this cardioprotection was directly linked to specific miRNAs present in MSC-EV that specifically targeted the cell death regulation machinery [96,97]. Collectively, these studies are of paramount importance because they suggest increased improvements in myocardial tissue survival by MSC-EV and how this beneficial effect plays a key role in preventing subsequent adverse remodeling once myocardial ischemia/reperfusion injury is critically established.

In vivo administration of MSC-EV could promote active processes of myocardial angiogenesis in ischemic hearts due to the high levels of proangiogenic factors that MSC-EV transfer locally. Following MSC-EV administration, EV-associated biomolecules trigger the proliferation and migration of endothelial-lineage progenitors or mature vascular cells. Hence, infarcted hearts treated with MSC-EV exhibited higher capillary densities compared to non-treated hearts within one month after myocardial/reperfusion injury [87,88,98,99].

Currently, over one hundred clinical studies using EV are registered in the database www.clinicaltrials.gov (accessed on 17 August 2021). The majority of these studies evaluate endogenous EV as blood biomarkers for diagnostics rather than therapy. Despite the promising observations from the above-mentioned pre-clinical experience, the number of experimental treatments based on MSC-EV reaching the clinical stage is still very scarce in the context of MI to date. To the best of our knowledge, only one clinical trial, which is devoted to the safety evaluation of intracoronary infusion of MSC-EV in patients with acute MI (Table 1), has been posted so far, particularly by Mayo Clinic’s investigators on 31 March 2020, without any patients recruited so far.

**Figure 2:**
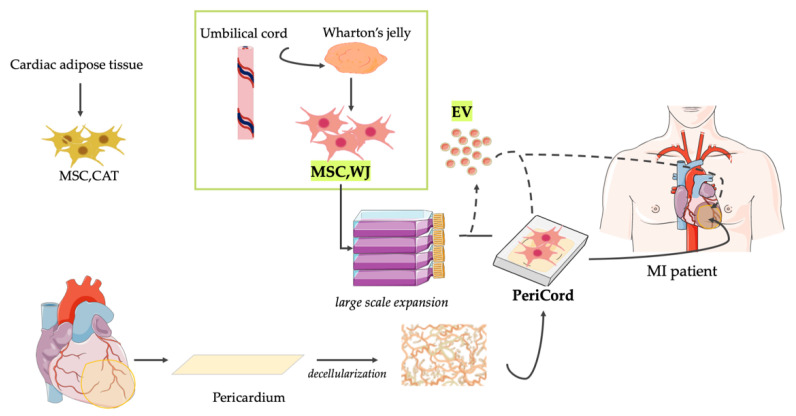
Current clinical translation of ATMP based on MSC and derived EV from Wharton’s jelly. For years, our laboratories have actively collaborated in exploring innovative treatments for MI. Particularly, our pre-clinical evidence-based experience includes the study and application of a variety of cell therapy and TE approaches using MSC derived from cardiac adipose tissue (MSC,CAT) and Wharton’s jelly (MSC,WJ). Recently, a clinical-size allogeneic cardiac bioimplant termed PeriCord has been implanted for limiting post-infarct sequelae in patients. As an alternative, the biomanufacturing and therapeutic use of novel ATMP based in MSC-EV resembling the characteristics of the parental MSC could be potentially adapted to the PeriCord production procedure. In fact, high yields of multifunctional EV with preserved function and purity could be isolated from the same large volume cultures of MSC,WJ prior to the generation of the PeriCord bioimplant. For that, however, progress in upcoming challenges, including good manufacturing practice and regulatory issues, will be crucial to demonstrate that this approach also holds potential for clinical translation. MSC,CAT = Cardiac Adipose Tissue-derived Mesenchymal Stromal Cells; MSC,WJ = Wharton’s jelly-derived Mesenchymal Stromal Cells; and EV = Extracellular Vesicles; MI = Myocardial Infarction.

Nevertheless, we apperceive that this is also the right time to advance the design, development and clinical translation of cell-free ATMP based on biomanufactured MSC-EV (Figure 2). For that purpose, it will be crucial to further: (i) comprehend their specific MoA; (ii) establish optimal dosing and dosage; (iii) better evaluate their biodistribution and potential adverse effects; and (iv) adhere to GMP quality management guidelines and regulatory requirements.

## 4. MSC-EV-Based Products: Clinical Perspectives and Biomanufacturing Challenges

Cell-based TE therapies have gained interest in the field of regenerative medicine as promising approaches for the repair of post-infarcted myocardial tissue. This foundation is based, in part, on the improvements to efficiently collect sample preparations enriched with high amounts of paracrine multifunctional factors, including multifunctional nanovesicles found in the conditioned medium from culture-expandable MSC. Notably, MSC-EV can induce phenotypic and epigenetic changes in neighboring cells while traveling long distances to transfer their specific molecular cargoes to targeted cells or tissues and modulate biological processes accordingly. For this, the scientific community envisions the versatility and clinical potential of MSC-EV as innovative, cell-free, immunomodulatory, pro-regenerative therapy approach post-MI. EV confer many advantages over the parental MSC themselves, as they: (i) are non-replicative biological, and thus their administration evades potential risks of tumorigenesis; and (ii) exhibit stable characteristics, including shelf-life, permeability, biodistribution and toxicity, over time in either the autologous or allogeneic setting indistinctly.

Regarding the translation of MSC-EV products into the clinics, MSC-EV biomanufacturing requires specialized facilities, skilled personnel and sufficient financial resources to first produce high amounts of the parental cells and then purify their released EV consistently, from batch to batch, in compliance with GMP procedures. EV isolation methods are still complex and involve the use of equipment not designed for this purpose. This most probably explains why treatments based on MSC-EV are poorly present in the clinical scenario to date. Their clinical use relies on open/semi-open production systems which are labor-intensive, require manual processing, and represent high investment in building classified environments, equipment and training skilled staff. Current open/semi-open systems for MSC culturing are also potentially associated with considerable increasing costs, risk of contamination, great variability across batches, and lack of real-time in-process control. For this, there has been a tendency toward the introduction of more sophisticated automated platforms, including scalable bioreactor systems, which may simplify the biomanufacturing workflow and optimize resources. These platforms could highly impact on robustness, traceability and yields of clinical-grade MSC expansion, also reducing production costs and allowing a number of in-process controls providing more accurate predictions of compliance with final product specifications.

Moreover, to achieve successful translation of MSC-EV into useful therapy candidates, MSC-EV processing has to resolve major concerns from a biomanufacturing perspective, including standardized in-process quality controls, identification of bioactive components in the cargo of EVs and potency testing, as well as further progress in instrumentation for optimal EV quantification and dosage. In this regard, EV yields remain limiting due to conventional MSC culture or microenvironment conditions, including cell density, aging and passage, stage of differentiation and substrate topography, which considerably affect their intrinsic properties. In this sense, the use of bioreactors with high cell growth surface, media recirculation and repeated supernatant recovery appears highly valuable to fulfil current clinical standards or requirements. On the contrary, animal-derived growth supplements are discouraging for clinical-grade MSC and derivatives biomanufacturing because they can potentially induce adverse clinical effects once therapeutic products are administered. Most of these supplements have undergone limited characterization, and they might harbor potential animal pathogens that remain critically unknown. Alternative chemically-defined MSC culture media formulations need also to be immediately addressed in forthcoming MSC-EV applications.

## Figures and Tables

**Table 1:** Current clinical trials with using MSC,WJ or EV in the treatment of cardiovascular diseases. * Reported results in [65].

Clinical Trial	Identifier	Abstract	N	Drug	Phase	State
Pericardial Matrix With Mesenchymal Stem Cells for the Treatment of Patients With Infarcted Myocardial Tissue (PERISCOPE) [50]	NCT03798353	Comparison of the outcome of patients who have undergone sternotomy to perform surgical revascularization and patients that, additionally, were implanted the PeriCord construct	Estimated: 12	Matrix-cell construct placed in the ischemic area (PeriCord)	1	Recruiting
Intracoronary Human Wharton’s jelly-derived Mesenchymal Stem Cells (MSC,WJ) Transfer in Patients With Acute Myocardial Infarction (AMI) (MSC,WJ-AMI) [65]	NCT01291329	Evaluation of safety and efficacy of MSC,WJ infusion in patients 4–7 days post-MI	116	MSC,WJ infusion or placebo	2	Completed *
Randomized Study of Coronary Revascularization Surgery With Injection of MSC,WJ and Placement of an Epicardial Extracellular Matrix (scorem-cells)	NCT04011059	Evaluation of the safety and effect of intramyocardial injection of MSC,WJ in coronary revascularization	Estimated: 40	MSC,WJ injection or placebo	1–2	Not yet recruiting
Intracoronary or Intravenous Infusion Human Wharton’s jelly-derived Mesenchymal Stromal Cells in Patients With Ischemic Cardiomyopathy (WJ-ICMP Tria)	NCT02368587	Evaluation of the safety and efficacy of MSC,WJ in patients suffering from ischemic cardiomyopathy secondary to MI	Estimated: 160	MSC,WJ infusion or placebo	2	Not yet recruiting
Cardiovascular Clinical Project to Evaluate the Regenerative Capacity of CardioCell in Patients With Acute Myocardial Infarction (AMI)	NCT03404063	Stablish a comparison of outcomes between patients suffering from ischemic damages treated with CardioCell and a control group (which will be receiving placebo)	105	Active IMP (known as CardioCell) and placebo	2–3	Completed
WJMSCs Anti-inflammatory Therapy in Coronary Artery Disease (WANICHD)	NCT04551456	Evaluation of the safety and anti-inflammatory efficacy of MSC,WJ in patients with coronary artery atherosclerosis disease	Estimated: 300	MSC,WJ infusion or placebo	2	Not yet recruiting
WJMSCs Anti-inflammatory Therapy in Acute Myocardial Infarction (WAIAMI)	NCT04551443	Evaluation of the safety and feasibility of WJMSCs in the treatment of patients in the acute phase (within 24 h) with the both of ST-Segment-Elevation or Non-ST-Segment-Elevation AMI.	Estimated: 200	MSC,WJ infusion or placebo		Not yet recruiting
Safety Evaluation of Intracoronary Infusion of Extracellular Vesicles in Patients With AMI	NCT04327635	Safety evaluation of EVs in treating patient with AMI	Estimated: 18	PEP drug (dosage of 5%; 10%; or 20%)	1	Recruiting
